# Consensual Coitus Leading to Vaginal Perforation, Bowel Evisceration, and Hemorrhagic Shock: A Report of a Rare Case

**DOI:** 10.7759/cureus.79015

**Published:** 2025-02-14

**Authors:** Hanane Houmaid, Karam Harou, Ahlam Bassir, Hamid Asmouki, Abderraouf Soummani

**Affiliations:** 1 Obstetrics and Gynecology Department, Mohammed VI University Hospital Center, Cadi Ayyad University, Marrakesh, MAR

**Keywords:** consensual intercourse, hemorrhagic shock, post-coital posterior fornix perforation, sexual education, sexual violence

## Abstract

Consensual sexual intercourse-induced vaginal rupture in adult women with no prior risk factors is a rare condition. We report the case of a 36-year-old nulliparous woman who presented in the gynecologic emergency department with post-coital posterior fornix perforation, evisceration, and profuse bleeding leading to hemorrhagic shock. It required urgent surgical management and hospitalization in intensive care.

Our ultimate goal is to highlight domestic sexual violence, which remains a taboo in our socio-cultural context, and to reiterate the importance of sexual education.

## Introduction

Intimate partner violence and sexual violence are highly associated with genital female tract injuries, a common reason for consultation in gynecologic emergency [1]. Percoital posterior vaginal fornix perforation communicating with the peritoneal cavity and bowel evisceration is a rare but serious event, and any delay in treatment can threaten the patient's life [2]. The first reported case in the literature was in 1978, following non-consensual intercourse [3].

In consensual sexual intercourse and especially of first timers, partners must be psychologically prepared and previously aware of their behaviors. Unfortunately, it is still taboo in our context to talk about sexuality, and in the lack of sexual education, wedding night can turn into a disaster. We report a case of consensual marital coitus complicated by posterior fornix perforation with evisceration and profuse bleeding leading to hemorrhagic shock requiring prompt resuscitation measures and surgical management, followed by hospitalization in intensive care.

## Case presentation

A 36-year-old virgin woman, with an unremarkable medical history, her last menstrual period being two weeks earlier, presented to the gynecologic emergency department with a one-hour history of acute abdominal pain and bright red vaginal bleeding, after her first consensual marital intercourse. She was accompanied by her husband, her mother, and her sister and denied any history of violence or vaginal instrumentation before the onset of symptoms.

However, when interviewed alone, she said she had been a victim of violence and forced penetration, with the use of fingers. She described her partner's penis as normal in size and anatomy and without deformity, but claimed he was may be under drug use effect, due to his aggressive and violent behaviors.

She was admitted for hemorrhagic shock, discolored conjunctiva with generalized abdominal defense, palpitation, and cold extremities. Her body temperature was 36.5°C, with an elevated pulse at 110 beats/minute, blood pressure was 95/60 mmHg, and respiratory rate was 18 breaths per minute. During the inspection of the perineum, we found exteriorized red bleeding and a recent lacerated hymenal tear, without rectovaginal injury. Rectal exam and sphincter tone were normal.

**Figure 1:**
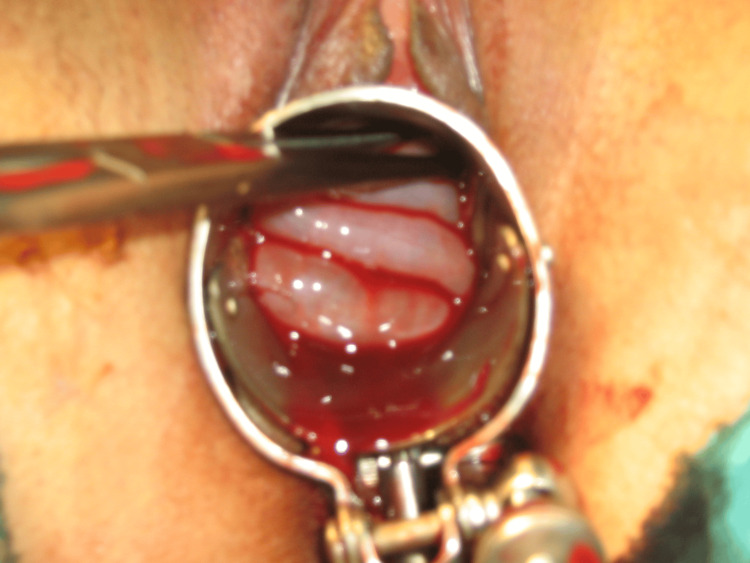
Perforation of the posterior cul-de-sac

**Figure 2:**
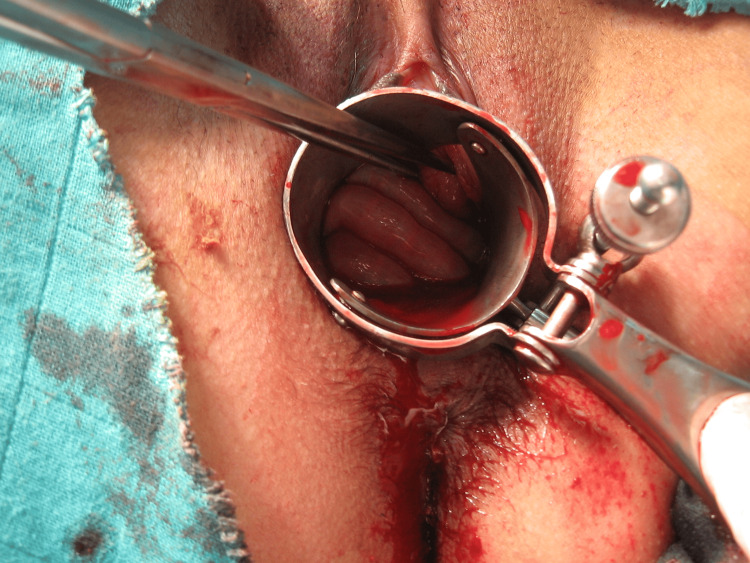
Herniation of viable small bowel

We urgently began resuscitation measures, and then we continued our examination under anesthesia for better evaluation. A speculum examination noted a 4-5 cm vaginal bleeding injury with perforation of the posterior cul-de-sac and herniation of viable small bowel (Figure 1 and Figure 2). Furthermore, the patient had a nulliparous's cervix without any lesion. A blood count showed hemoglobin at 6 g/dl, and platelets, white blood cell count, and prothrombin time were within the normal range. The patient benefited from prompt intensive care management and gynecologic surgery. The vaginal rupture was sutured by vaginal route in a continuous, full-thickness layer with 2-0 chromic catgut, and the hemostasis was assured. The patient received antibiotic prophylaxis, four packs of red blood cells, and six bags of fresh frozen plasma and spent two days in intensive care.

The patient's postoperative course was uneventful, she was discharged on day 3, and she was advised to abstain from sexual intercourse for 10-15 weeks with possible psychiatric care.

## Discussion

Sexual intercourse is the most common cause of lesions of the lower genital tract in women, in addition to obstetric causes [4]. They can range from superficial vaginal lacerations that can resolve spontaneously or require simple care to deeper and more serious lacerations. Sometimes, it can even cause perforations extending into the peritoneal cavity or the rectal lumen and can be life-threatening requiring heavy management [5,6]. This situation is exceptional, because the pelvic anatomy and physiology are appropriate to consensual intercourse.

Successful intercourse especially for the first time needs a previous good sexual education for both partners and psychological preparation. Moreover, at the moment of sexual relations, physiological conditions are mandatory. In fact, as part of sexual arousal, the vagina becomes lubricated and increases in dimensions to facilitate penile penetration; also, the uterus and cervix elevate within the pelvic cavity [7]. Sexual intercourse that occurs without respecting these conditions is more likely to result in damage to the genital tract.

Around 49-100% of percoital injuries have been reported in virgin women [2]. Several factors may predispose to genital tract injuries such as extremes of age, first coitus, non-consensual and forced sex, female position, female-male genital disproportion size, use of foreign objects or fingers, use of aphrodisiacs, rushed sexual intercourse, sex under the influence of drugs, inexperience, vaginismus, pregnancy, retroversion of the uterus, a long period of abstinence, previous surgery, and congenital anomalies [4,8]. However, in many cases, the true mechanism of injuries is often difficult to know. Indeed, it's strongly hard and embarrassing for the patient and her partner to reveal their erotic practices, so they prefer to deny any insertion of sex toys or other objects if there is any. Moreover, the woman didn't admit she underwent domestic aggressive sexual behavior and that she was a victim of sexual violence.

Consensual coitarche is generally accompanied by bleeding and often minimal injuries requiring no medical advice. Some cases of life-threatening hemorrhagic shock, evisceration, post-coital posterior fornix perforation, and peritonitis due to intense and forceful sexual penetration have been reported [9,10].

The most common injury location is the posterior portion of the right fornix, resulting from the uterus lying in an anteverted position, slightly to the right in the majority of women [11].

Several cases of posterior fornix perforation associated with evisceration have been described in the literature. It is a rare medical condition, especially in women without risk factors like vaginal atrophy and a history of pelvic surgery most often hysterectomy, but it remains a serious surgical emergency [1,12]. Delays in medical consultation, diagnosis, or management can be life-threatening. Indeed, the patient could be exposed to the risk of hemorrhagic shock, fatal air embolism, peritonitis, hollow organ perforation, bowel occlusion, and ischemia [2,13,14].

The treatment of the vaginal perforation is always surgical, although several approaches have been described. There is no consensus on the appropriate method of repair for cuff laceration. However, various surgical approaches have been reported in the literature, either laparoscopic, vaginal, or laparotomic approaches [15].

Psychosexual support is also very important to help those women in order to move on in their intimate lives.

To prevent the traumas caused by sexual intercourse and their complications, both partners must have at least a sexual education. Some anatomic conditions seem to be difficult to overcome, like disproportionate genital size and the position of uteri, but it's advised to consult a specialist in sexology or psychosexology.

## Conclusions

This case is reported to highlight domestic sexual violence, which remains a taboo in our socio-cultural context. Our ultimate goal is to reiterate the importance of early diagnosis and prompt multidisciplinary management to avoid complications which could be life-threatening. Prevention by good sexual education remains the gold standard management to reduce the morbidity and mortality of this medical entity.
